# Psychiatric staff on the wards does not share attitudes on aggression

**DOI:** 10.1186/1752-4458-8-14

**Published:** 2014-04-22

**Authors:** Tero Laiho, Nina Lindberg, Grigori Joffe, Hanna Putkonen, Anja Hottinen, Raija Kontio, Eila Sailas

**Affiliations:** 1 Department of Psychiatry, Helsinki University Central Hospital, Helsinki, Finland; 2 Hospital District of Helsinki and Uusimaa, Hyvinkää Hospital Area, Tuusula, Finland; 3 Faculty of Medicine, Forensic Psychiatry, University of Helsinki, Helsinki, Finland; 4 Vanha Vaasa Hospital, Vaasa, Finland

**Keywords:** Aggression, Attitude, Staff, Ward culture

## Abstract

**Background:**

The concept of ward culture has been proposed as a reason for the often reported differences in treatment decisions when managing inpatient aggression. We therefore studied whether staff on wards actually shares similar perceptions and attitudes about aggression and whether the specialty of the ward on which the staff members work influences these opinions.

**Methods:**

The Attitudes Towards Aggression Scale was used to assess attitudes towards aggression in 31 closed psychiatric wards. Altogether 487 staff members working on the study wards were asked to fill in the scale. Respondent’s gender, age, educational level, working experience on the current ward, and specialty of this ward (acute, forensic, rehabilitation) served as background variables.

**Results:**

Most of the variance found was due to differences between individuals. Belonging to the personnel of a particular ward did not explain much of the variance.

**Conclusions:**

Psychiatric staff on the wards does not share attitudes on aggression. As each staff member has his/her own opinion about aggression, training for dealing with aggression or violent incidents should be done, at least partly, on an individual level. We also suggest caution in using the concept of ward culture as an explanation for the use of restrictive measures on psychiatric wards.

## Background

Psychiatric inpatients exhibiting aggressive behavior towards staff or fellow patients are a challenge for clinical management [1]. The proportion of patients acting aggressively during their stay on acute psychiatric wards varies between 8% and 44% [2]. Being subjected to verbal abuse or violent behavior can result in psychological trauma in addition to possible physical injury [3]. Coercive interventions, such as seclusion and mechanical restraint, are common methods for managing violent behavior during psychiatric hospitalization. Their use is highly controversial as they restrict the patient’s freedom, being used against his/her will [4]. Available data suggest that marked differences both in numbers of patients subjected to various coercive interventions and in durations of these interventions between countries [5]. Similar differences have been reported between different treatment wards [6]. This can be only partly explained by patient compilation [7] and the physical environment of the psychiatric wards [8]. Nurses have reported that the decision-making process for managing patients’ aggressive behavior poses inherent ethical dilemmas [9].

Some studies have stressed the importance of organizational factors in the treatment of inpatient aggression alone [10] or in combination with staff-level factors [11]. In discussions on the rationale of treatment decisions on the ward, references are frequently made to the concept of ward culture [12]. Holland [13] reported that a cultural system in society could be identified by five major components: the environment in which the group functions, the material environment used by the group, a common cultural tradition, human activities, and behaviors emerging from the complex interactions of these four components. Thus, according to Holland, each ward has its own culture with particular rituals. Other definitions of ward culture are the internalized assumptions, attitudes, understandings, and beliefs commonly held by clinicians on the unit that guide interaction styles and clinical decision-making [14] or simply “the way things are done around here” [15]. Ward culture has also been seen as a part of the context in which treatment is given and in which different reforms are implemented [16]. It has been described as something shared and accepted, and behaviors are both prescribed and proscribed. It involves tacit knowledge and new members are supposed to absorb non-articulated group viewpoints [17].

### Aim of the study

The concept of ward culture has been proposed as a reason for the often reported differences in treatment decisions when managing inpatient aggression. We therefore investigated whether staff on wards actually shares similar perceptions and attitudes about aggression and whether the specialty of the ward on which the staff members work influences these opinions.

## Methods

### Study design

This cross-sectional study was conducted in Finland as a part of SAKURA, an extensive research and development project to investigate and reduce the use of seclusion and restraint in Finland and in Japan [18]. It comprised all closed adult wards in three major psychiatric hospitals in southern Finland. Four wards were excluded as they were involved in other research and development projects. The specialist care provided on the study wards (n = 31) was Acute (n = 13), Forensic (n = 7), and Rehabilitation (n = 11). The total number of staff working on the wards was 487. In order to study homogeneity of attitudes generalized to ward level, wards with response rate > 50% were selected for further analysis.

### Instrument

The Attitudes Towards Aggression Scale (ATAS) was used to study individual attitudes towards aggression [19]. This 18-item scale comprises statements concerning different aspects of aggression. Every statement is given a Likert-type scale ranging from strongly agree (value 5), to strongly disagree (value 1). Thus, the sum score of the scale ranges from 18 to 90. In our study, the ATAS was translated to Finnish and back- translated to English by the members of the research group.

The ATAS consists of five aggression- related components: *offensive* (unpleasant and unacceptable behavior; statements: aggression 1. is destructive behavior and therefore unwanted, 2. is unnecessary and unacceptable behavior, 3. is unpleasant and repulsive behavior, 4. is an example of a non-cooperative attitude, 5. poisons the atmosphere on the ward and obstructs treatment, 6. in any form is always negative and unacceptable, 7. cannot be tolerated), *communicative* (in the sense of signals to enhance the therapeutic relationship; statements: aggression 1. offers new possibilities in nursing care, 2. helps the nurse to see the patient from another point of view, 3. is the start of a more positive nurse relationship), *destructive* (in the form of actual harmful acts; statements: aggression 1. is when a patient has feelings that will result in physical harm to self or to others, 2. is violent behavior to others or self, 3. is threatening to damage others or objects), *protective* (the defense of physical and emotional space; statements: aggression 1. is to protect oneself, 2. is the protection of one’s own territory and privacy), and *intrusive* (the intention to damage or injure others; statements: aggression 1. is a powerful, mistaken, non-adaptive, verbal and/or physical action done out of self-interest, 2. is expressed deliberately, with the exception of aggressive behavior of someone who is psychotic, 3. is an impulse to disturb and interfere in order to dominate or harm others).

The ATAS is regarded as a theoretically conclusive scale that can be used to ascertain differences in attitudes among groups. Earlier research using the ATAS has reported significant differences in attitudes in relation to aggression linked to personnel’s gender [20] and to frequencies in the use of de-escalation or restrictive coercive measures to manage aggression on wards [21]. In this study, respondent’s gender, age, educational level, working experience on the current ward, and specialty of this ward (acute, forensic, rehabilitation) served as background variables.

### Data collection

The data were collected in May 2008. Information about the research was given at nurse managers’ meetings and staff meetings. The questionnaires for the nursing staff were distributed to the wards by three of the researchers, and the questionnaires for physicians were distributed during clinical meetings. The questionnaires were distributed to all of the staff on the wards. To ensure respondents’ anonymity, the wards were supplied with return envelopes and closed containers. After the data collection period, the containers were removed by the researchers.

### Statistical procedures

The validity and reliability of the ATAS scale were assessed with Principal Component Analysis with Oblimin rotation and Cronbach’s Alpha correlation. For each aggression-related component, a sum variable was created and these were used for further analyses. Variance analysis was performed using One-way Analysis of Variance (ANOVA). Mean scores of components and standard deviation of means were also calculated.

### Ethical considerations

The study protocol for the whole SAKURA research project was duly approved by the Ethics Committee of the Hospital District of Helsinki and Uusimaa. The appropriate authorities in each participating hospital granted separate research permission. The questionnaire was used with the permission of the developer. The participants received oral and written information on the purpose of the study and their rights as respondents. Participation in the study was voluntary and the respondent’s anonymity was ensured in all phases of data collection and analysis.

## Results

Of the 487 questionnaires distributed, 397 were returned; thus the overall response rate was 81.5%. The ATAS was completed in 361 cases (91.0%). Respondents’ background data are provided in Table 1. Of 31 wards, 15 (48%) (acute: n = 7, forensic: n = 4, rehabilitation: n = 4) exhibited a response rate > 50%.

**Table 1:** Background variables of 361 staff members who filled in the attitudes towards aggression scale (ATAS)

**Gender**	**n**	**%**
Female	228	63.2
Male	125	34.6
Missing information	8	2.2
**Age**		
< 30 years	74	20.5
30-39 years	108	29.9
40-49 years	109	30.2
≥ 50 years	64	17.7
Missing information	4	1.7
**Educational level**		
Psychiatric nurse (registered)	145	40.2
Mental health nurse (licensed)	166	45.9
Psychiatrist	17	4.7
Missing information	33	9.1
**Specialty of ward**		
Acute psychiatry	135	37.4
Forensic psychiatry	49	13.6
Psychiatric rehabilitation	51	14.1
Missing information	126	34.9
**Working experience on current ward**		
< 2 years	103	28.5
2-5 years	109	30.2
6-10 years	57	15.8
≥ 11 years	75	20.8
Missing information	17	4.7

The reliability of the scale was good (Cronbach’s alpha 0.70). Barlett’s test of sphericity was conducted, with observed p < 0.001. The KMO sampling adequacy was 0.783, exceeding the recommended value of 0.6 [22].

Using Principal Component Analysis with Oblimin rotation, the items formed five component structures, as in earlier ATAS studies conducted in Europe [19,20]. Component structure accounted for 65.9% of the variance. Correlation between components was also found to be identical with the original research, with a positive correlation between protective and communicative components (0.25), and between offensive and intrusive components (0.25). Negative correlations were found between offensive and communicative components (−0.23) and between offensive and protective components (−0.21).

**Figure 1:**
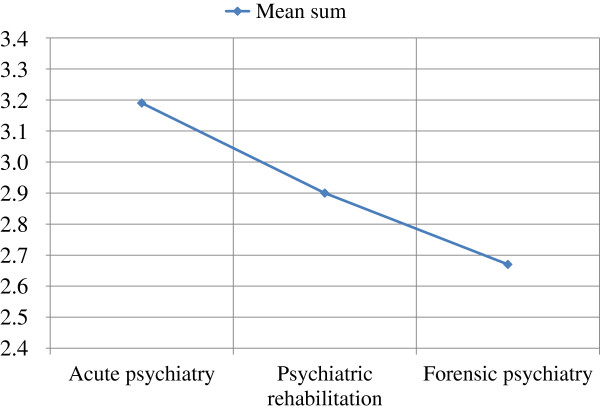
Mean sum of Protective component in different specialties (acute [n = 13], rehabilitation [n = 11], and forensic psychiatry [n = 7]).

**Figure 2:**
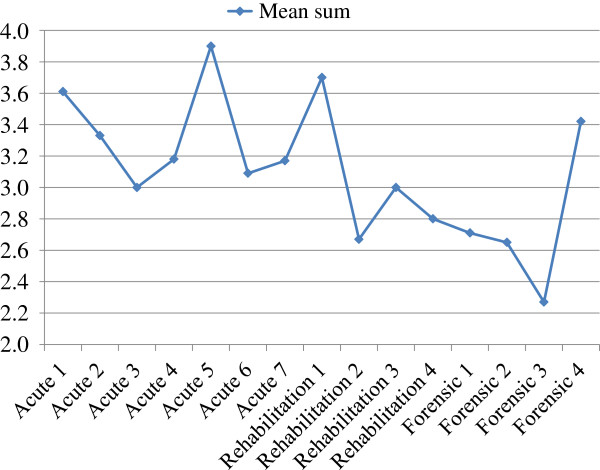
Mean sum of Protective component in wards with response rate > 50% (n = 15).

Most of the variance in attitudes towards aggression seemed to be due to differences between individuals rather than the result of belonging to a group. Using variance analysis, most of the variance found was not related to respondents’ gender, age, educational level, working experience, or specialty of the ward in which the respondent worked (see Table 2). Only two statistically significant associations emerged. First, the offensive component was associated with respondent’s working experience on the current ward. However, the clinical relevance of this finding is arguable, as the means are quite congruent and the range from 3.13 to 3.48 has overlapping confidence intervals (see Table 3). Second, significant differences among specialties were observed on the protective component (see Table 2 and Figure 1). In contrast to the first result, this finding might have clinical relevance, as the difference of means between acute and forensic psychiatry is 0.51 (acute psychiatry: 3.18, forensic psychiatry: 2.67) (Table 3). The significance remained when differences among wards with response rate > 50% were considered, showing predominance of the protective component on acute wards versus forensic wards (see Table 2 and Figure 2). Although psychiatric rehabilitation did not differ significantly from acute or forensic psychiatry on the protective component, the position of psychiatric rehabilitation between acute and forensic wards is to be acknowledged.

**Table 2:** Variance in background variables of 361 staff members who filled in the attitudes towards aggression scale (ATAS)

**Age**	**Sum of squares**	**Mean square**	**df**	**F**	**p**
ATAS component: Offensive	4.15	1.38	3	1.551	0.201
Communicative	3.26	1.09	3	1.539	0.204
Destructive	0.45	0.15	3	0.275	0.844
Protective	0.75	0.25	3	2.231	0.875
Intrusive	0.49	0.16	3	0.163	0.880
**Gender**					
Offensive	1.78	1.78	1	1.986	0.160
Communicative	2.51	2.51	1	3.550	0.060
Destructive	0.18	0.18	1	0.333	0.564
Protective	0.74	0.74	1	0.069	0.793
Intrusive	0.01	0.01	1	0.003	0.956
**Educational level**					
Offensive	1.97	0.99	2	1.129	0.325
Communicative	1.42	0.71	2	0.981	0.376
Destructive	0.62	0.31	2	0.581	0.560
Protective	0.42	0.21	2	0.198	0.820
Intrusive	1.3	0.64	2	0.886	0.413
**Working experience on current ward**					
Offensive	7.20	2.40	3	2.710	**0.045**
Communicative	1.15	0.38	3	0.538	0.657
Destructive	2.01	0.67	3	1.231	0.298
Protective	3.12	1.04	3	0.959	0.412
Intrusive	3.99	1.33	3	1.846	0.139
**Specialty of ward**					
Offensive	2.77	1.39	2	1.460	0.234
Communicative	1.92	0.96	2	1.277	0.281
Destructive	0.41	0.21	2	0.388	0.679
Protective	10.23	5.11	2	4.710	**0.010**
Intrusive	2.28	1.14	2	1.523	0.220
**Wards with response rate > 50%**					
Offensive	12.00	0.80	15	0.90	0.567
Communicative	8.71	0.58	15	0.76	0.716
Destructive	5.63	0.38	15	0.69	0.792
Protective	32.35	2.16	15	2.10	**0.012**
Intrusive	10.95	0.73	15	1.02	0.439

One-Way Analysis of Variance (ANOVA) was used.

**Table 3:** The attitudes towards aggression scale (ATAS) component scores (mean, SD) and their confidence intervals related to background variables

**Gender**		**Mean**	**SD**	**95% Cl**
ATAS component: Offensive	Female	3.24	0.94	3.12 - 3.37
	Male	3.39	0.94	3.22 - 3.56
	Total	3.30	0.94	3.19 – 3.40
Communicative	Female	2.23	0.81	2.12 – 2.34
	Male	2.41	0.89	2.25 – 2.57
	Total	2.29	0.84	2.20 – 2.38
Destructive	Female	4.18	0.72	4.08 – 4.28
	Male	4.13	0.76	4.00 – 4.27
	Total	4.16	0.74	4.08 – 4.24
Protective	Female	3.00	1.05	2.86 – 3.14
	Male	3.03	1.01	2.85 – 3.21
	Total	3.01	1.03	2.90 – 3.12
Intrusive	Female	3.48	0.84	3.37 – 3.59
	Male	3.49	0.86	3.33 – 3.64
	Total	3.48	0.85	3.39 – 3.57
**Age**				
Offensive	< 30	3.09	0.90	2.88 – 3.30
	30-39	3.34	0.96	3.16 – 3.53
	40-49	3.35	0.93	3.17 – 3.53
	≥ 50	3.40	0.95	3.14 – 3.65
	Total	3.30	0.95	3.20 – 3.40
Communicative	< 30	2.37	0.72	2.20 – 2.54
	30-39	2.15	0.88	1.97 – 2.32
	40-49	2.30	0.83	2.14 – 2.47
	≥ 50	2.40	0.92	2.16 – 2.63
	Total	2.29	0.84	2.20 – 2.38
Destructive	< 30	4.12	0.63	3.97 – 4.27
	30-39	4.21	0.73	4.07 – 4.36
	40-49	4.14	0.82	3.99 – 4.30
	≥ 50	4.17	0.72	3.99 – 4.35
	Total	4.17	0.73	4.09 – 4.24
Protective	< 30	3.03	0.91	2.82 – 3.25
	30-39	2.95	1.05	2.75 – 3.16
	40-49	3.06	1.11	2.85 – 3.28
	≥ 50	2.98	1.04	2.71 – 3.24
	Total	3.01	1.04	2.90 – 3.12
Intrusive	< 30	3.45	0.79	3.26 – 3.63
	30-39	3.45	0.79	3.30 – 3.60
	40-49	3.52	0.95	3.33 – 3.70
	≥ 50	3.53	0.87	3.31 – 3.75
	Total	3.48	0.85	3.39 – 3.58
**Educational level**				
Offensive	Nurses (reg.)	3.24	0.90	3.09 – 3.39
	Nurses (lic.)	3.32	0.98	3.17 – 3.48
	Psychiatrists	2.99	0.70	2.62 – 3.34
	Total	3.27	0.93	3.17 – 3.37
Communicative	Nurses (reg.)	2.25	0.82	2.11 – 2.39
	Nurses (lic.)	2.36	0.89	2.22 – 2.50
	Psychiatrists	2.10	0.71	1.73 – 2.48
	Total	2.30	0.85	2.20 – 2.39
Destructive	Nurses (reg.)	4.12	0.78	3.99 – 4.25
	Nurses (lic.)	4.21	0.69	4.10 – 4.31
	Psychiatrists	4.12	0.63	3.79 – 4.44
	Total	4.16	0.73	4.08 – 4.24
Protective	Nurses (reg.)	3.05	1.02	2.88 – 3.22
	Nurses (lic.)	3.00	1.05	2.84 – 3.16
	Psychiatrists	2.91	1.02	2.36 – 3.45
	Total	3.02	1.03	2.91 – 3.13
Intrusive	Nurses (reg.)	3.46	0.84	3.32 – 3.60
	Nurses (lic.)	3.55	0.85	3.41 – 3.68
	Psychiatrists	3.29	0.93	2.80 – 3.79
	Total	3.49	0.85	3.40 – 3.59
**Working experience on current ward**				
Offensive	< 2 years	3.13	0.98	2.93 – 3.32
	2-5 years	3.23	0.93	3.04 – 3.41
	6-10 years	3.48	0.81	3.25 – 3.70
	≥ 11 years	3.46	0.95	3.24 – 3.69
	Total	3.29	0.95	3.19 – 3.39
Communicative	< 2 years	2.30	0.80	2.14 – 2.46
	2-5 years	2.36	0.89	2.18 – 2.53
	6-10 years	2.30	0.84	2.07 – 2.53
	≥ 11 years	2.19	0.85	2.00 – 2.39
	Total	2.29	0.84	2.20 – 2.39
Destructive	< 2 years	4.13	0.75	3.98 – 4.28
	2-5 years	4.18	0.70	4.04 – 4.31
	6-10 years	4.33	0.65	4.15 – 4.50
	≥ 11 years	4.09	0.83	3.90 – 4.28
	Total	4.17	0.74	4.09 – 4.25
Protective	< 2 years	2.94	1.06	2.73 – 3.16
	2-5 years	3.15	0.99	2.96 – 3.34
	6-10 years	2.91	1.09	2.61 – 3.21
	≥ 11 years	2.99	1.05	2.75 – 3.23
	Total	3.01	1.04	2.90 – 3.13
Intrusive	< 2 years	3.46	0.83	3.30 – 3.63
	2-5 years	3.35	0.85	3.18 – 3.51
	6-10 years	3.58	0.88	3.33 – 3.83
	≥ 11 years	3.62	0.85	3.43 – 3.82
	Total	3.48	0.85	3.39 – 3.57
**Specialty of ward**				
Offensive	Acute	3.19	1.00	3.01 – 3.36
	Rehabilitation	3.34	0.96	3.07 – 3.62
	Forensic	3.45	0.91	3.19 – 3.71
	Total	3.28	0.96	3.15 – 3.41
Communicative	Acute	2.21	0.81	2.07 – 2.36
	Rehabilitation	2.36	0.92	2.10 – 2.62
	Forensic	2.43	0.94	2.16 – 2.70
	Total	2.29	0.87	2.18 – 2.41
Destructive	Acute	4.17	0.73	4.04 – 4.30
	Rehabilitation	4.07	0.84	3.83 – 4.31
	Forensic	4.18	0.60	4.00 – 4.35
	Total	4.15	0.73	4.05 – 4.24
Protective	Acute	3.18	1.03	3.01 – 3.36
	Rehabilitation	2.90	1.06	2.60 – 3.20
	Forensic	2.67	1.04	2.37 – 2.97
	Total	3.01	1.06	2.88 – 3.15
Intrusive	Acute	3.39	0.93	3.23 - 3.55
	Rehabilitation	3.41	0.79	3.19 – 3.64
	Forensic	3.65	0.76	3.42 – 3.87
	Total	3.45	0.87	3.33 – 3.56

Of all components, the destructive component showed the least variance among different wards and individuals. This suggests that, regardless of background variables or working environment, aggression is almost invariably seen as destructive behavior (mean 3.35, median 3, SD 0.54).

## Discussion

We found the ATAS to be a useful instrument for studying personnel’s attitudes towards aggression. In contrast to earlier research [20], the positions on aggression varied widely and the variation could not be explained by respondents’ gender, age, educational level, working experience on the current ward, or specialty of the ward – with a few exceptions. Our respondents were virtually unanimous regarding aggression being seen as concrete actions and having a destructive component. As this is a very common definition of aggression, the finding is not surprising. Seeing aggression as having protective dimensions was related to the working environment and to the type of patients. On the other hand, emphasizing the offensive and intrusive components of aggression was not related to working environment, the type of patients treated, or the gender, age, or working experience of the respondent. These opinions about aggression could not be explained by any variable other than respondents’ individual ways of thinking. Thus, they seem to reflect the individual opinions of the staff.

Respondents working on acute psychiatric wards, and to a lesser extent respondents working on rehabilitation wards, emphasized the protective component of aggression, unlike those working in forensic psychiatry. This could be explained as a reaction to having witnessed the instrumental violence that occurs more often on forensic wards than on acute or rehabilitation wards [23]. Also, on acute wards, occasional violent episodes can easily be attributed to the distorting experience of psychosis. On the other hand, on forensic wards, and to a lesser extent on rehabilitation wards, periodic threat of repeated violence exists. On different wards within one area of specialization, differences also emerged in recognizing of the protective component of aggression. As the differences were substantial, we do not consider this a random finding, but can offer no evident explanation for it.

Working experience on the current ward predicted how much the offensive component of aggression was stressed. The longer a respondent had worked on the current ward, the more he/she highlightened the offensiveness of aggression. The reason for this might be that a longer career usually means that an individual has witnessed more violence on a ward. On the other hand, a long career could also result in becoming inured to the communicative and protective features of aggression.

In summary, each individual seems to have his/her personal thoughts, views, and attitudes on aggression. Thus, we challenge the concept of ward culture if defined as shared attitudes. People on the same ward tend to work similarly, but this is related more to shared rituals, rules, and management than to socialized thinking. To revise outdated or questionable treatment practices, there is a need for personalized training, management, tutoring, and reflection.

We also suggest that the components of the ATAS scale – and the results of this study – are associated with three separate dimensions. The first dimension comprises the idea that aggression can be seen as concrete destructive actions, a widely and uniformly shared opinion among personnel and in society. The second dimension consists of communicative and protective components that highly correlate with each other and represent the “understanding view” of aggression. This dimension seems to be triggered by patients and/or wards, and thus, one could speculate that the understanding of aggression is something related to patients and ward culture. The third dimension represents facets of aggression as dysfunctional behavior and consists of offensive and intrusive components that are interrelated. In the present study, this dimension seemed not to be linked to any known background information, and therefore, it might be related to individual life experiences and arise from respondents’ personal histories or experiences.

### Strengths and limitations

The strength of this study was the large number of psychiatric wards and staff members. The participation rate can be regarded as reasonable. Unfortunately, almost 35% of the respondents did not provide information about the specialty of his/her ward. From this perspective, the study must be regarded as preliminary.

## Conclusions

Psychiatric staff on the wards does not share attitudes on aggression. As each staff member has an own individual opinion about aggression, training for dealing with aggressive or violent incidents should be done, at least partly, on an individual level. We also suggest caution about using the concept of ward culture as an explanation for the use of restrictive measures on psychiatric wards.

## Competing interests

The authors declare no competing interests.

## Authors’ contributions

TL, ES, HP, and GJ designed the study. TL, ES, and NL collected the data. TL performed the statistical procedures. All authors contributed to the analysis of the data. TL, ES, and NL drafted the manuscript. All authors revised the manuscript and read and approved the final version.
